# Gunshot Wounds of the Abdomen

**Published:** 1900-01-06

**Authors:** 


### GUNSHOT WOUNDS OF THE ABDOMEN.

PERHAPS the most interesting surgical item from the seat of war is the statement made by Mr. Makins in a letter to the *British Medical Journal* in regard to wounds of the abdomen. He says that he has seen 14 of these cases, 10 of which have exhibited no serious symptoms, and will probably get well. The pulses have not risen above 80, and the only signs have been some local tenderness, rigidity, and deficient mobility of the belly. All came down to Orange River on the third day after the injury. Slight vomiting occurred in some of them before arrival. In two cases the injury probably implicated the kidney, and in one the liver, but in all three the hæmorrhage must have been very slight. In four other cases, however, peritoneal infection had already occurred, and in three of these abdominal section was done, but only one of them did well. "All that can be said from this experience is," says Mr. Makins, "that no patient should be operated upon from the mere fact of apparent traverse of the belly by a Mauser or Lee-Metford bullet." It is worth while to compare with this statement the conclusions which were insisted on by some of the speakers at the recent discussion at Portsmouth on gunshot wounds of the abdomen. Colonel Stevenson, R.A.M.C., said, "When a projectile traversed the abdominal cavity we may rely, with practical certainty, on the occurrence of one or both of two very serious conditions—the extravasation of the intestinal contents, or a hæmorrhage which will not cease until mechanical means are employed to control it. In the latter case death may be looked for at an early period, and in the former at one but little more remote, but the fatal issue is almost certain in either. With these premises granted—and few surgeons will, in these days, deny them—the indications for the treatment are quite distinct; the abdomen must be opened, the hæmorrhage treated by the usual means, the perforations in the intestines closed by sutures, and the peritoneum cleansed." Further on he says, "It is indeed almost waste of time and words to insist on the great probability of visceral lesion and extravasation when a bullet is known to have traversed the abdomen . . . . I agree with Sir William MacCormac, who says, 'the bowel is nearly always perforated, fæces are extravasated, and very frequently there is some other complication as well;' that, in fact, once penetration of the abdomen by a bullet has been clearly ascertained, we may rely on the existence of visceral lesion; and when the intestinal tube is opened, even in the smallest degree, we may assume extravasation of its contents and peritonitis as certain to occur. The deduction to be made from this is self-evident—that all penetrating gunshot wounds of the abdomen should be treated by immediate laparotomy." The same has been taught by Nussbaum, Trelat, Greig Smith, Lucas, MacCormac, and many others. Evidently the present war gives promise of much new surgical teaching.

